# Tumor regression is mediated via the induction of HER2_63-71_- specific CD8+ CTL activity in a 4T1.2/HER2 tumor model: no involvement of CD80 in tumor control

**DOI:** 10.18632/oncotarget.15816

**Published:** 2017-03-01

**Authors:** Sayyed Nilofar Danishmalik, Si-Hyeong Lee, Jeong-Im Sin

**Affiliations:** ^1^ BK21 Plus Graduate Program and Department of Microbiology, School of Medicine, Kangwon National University, Chuncheon, Gangwon-do 200-701, Korea

**Keywords:** antitumor immunity, CD80, MDSCs, HER2, tumor models

## Abstract

In the CT26/HER2 and 4T1.2/HER2 tumor models, CT26/HER2 cells form tumors that continue to grow, whereas 4T1.2/HER2 cells form tumors that eventually regress. Here, we investigated the differences in the behaviors of these two cell lines. When immune cells from 4T1.2/HER2 tumor-bearing animals were stimulated with HER2 class I peptides, they displayed a 2-fold increase in IFN-γ levels, in response to the peptides, HER2_63–71_ and HER2_342–350_. In contrast, extremely high levels of antigen-non-specific IFN-γ production were observed with immune cells and sera from CT26/HER2 tumor-bearing mice. However, IFN-γ had no effect on tumor progression in the CT26/HER2 model, as determined by an IFN-γ knockout assay. 4T1.2/HER2 tumor-bearing mice displayed CTL activity in response to HER_263–71_ but not to HER2_342–350_, whereas no such induction was observed in CT26/HER2 tumor-bearing mice. When 4T1.2/HER2 cell-challenged mice were depleted of CD8+ T cells, they lost their tumor-regressing activity, suggesting an antitumor role of HER2_63–71_-specific CD8+ CTLs in the control of this tumor type. CT26/HER2 cells also expressed CD80. However, CD80-transfected 4T1.2/HER2 and CD80-non-expressing CT26/HER2 cells failed to alter their tumorigenicity, suggesting no role of CD80 in tumor control. Despite increased levels of myeloid-derived suppressor cells in the tumor, they were not associated with tumor progression in the CT26/HER2 model, as determined by a cell depletion assay. Overall, these data show that, contrary to CT26/HER2 tumors, 4T1.2/HER2 tumors regress via the induction of HER2_63–71_-specific CD8+ CTLs and that CD80 is not associated with the regression of these tumors.

## INTRODUCTION

Human epidermal growth factor receptor 2 [HER2] (also termed Her-2/neu and erbB-2) proteins are a type I family of epidermal growth factor receptors expressed on the cell surface [1, 2]; these proteins are over-expressed in approximately 30% of breast cancers [3]. They are also over-expressed in ovarian tumors and colorectal malignancies [4]. To date, ligands for erbB-2 proteins are still not defined. Clinically, the overexpression of erbB-2 is correlated with poor survival and short time to relapse rates in breast cancer patients [3]. ErbB-2 overexpression in human breast cancer cells also promotes their metastatic potential [5]. ErbB-2-specific antibodies and T cells are detectable in patients with breast and ovarian cancers [6, 7], suggesting that erbB-2 could be a target for immunotherapy. In this regard, Herceptin (anti-erbB-2 Ab) has been clinically available for use in improving therapeutic goals in patients with breast cancers that overexpress erbB-2 proteins [8, 9]. However, cancer relapse in spite of antibody treatment and resistance to antibody treatment in metastatic settings remain major clinical problems for many HER2-positive breast cancer patients [10].

Numerous clinical trials using HER2-targeting therapeutic vaccines (e.g., peptide vaccines, protein vaccines, DNA vaccines and cell type vaccines) have been tested for their therapeutic outcomes in breast cancer patients (reviewed in [11]). For instance, the administration of HLA-A2/3-restricted HER2 peptide (369–377) plus GM-CSF to patients with HER2-positive breast cancer following the completion of standard courses of therapy showed a 98.7% 5-year disease-free survival rate compared with an 80.2% rate for control patients (*p* = 0.08) [12], suggesting that the vaccine regimen might have some modest effect in preventing disease recurrence. Similarly, HER2-based vaccination approaches have been well studied in numerous animal model systems, such as mouse mammary D2F2 cells expressing HER2 [13], mouse colon CT26 cells expressing human erbB-2 (HER2) [14, 15], mouse thymoma EL40 cells expressing HER2 [16] and TUBO cells (rat neu transplantable mouse mammary carcinoma cells from BALB-rat neu transgenic mice) [17]. In particular, HER2 DNA vaccines have been shown to induce Ag-specific CD8+ CTL lytic activity against CT26/HER2 cells and antitumor prophylactic responses to a tumor cell challenge [14]. More recently, Foy et al. [15] reported that a combination of HER2-targeting active immunotherapy and anti-CTLA-4 antibody therapy increased survival rates from a metastatic CT26/HER2 tumor cell challenge by improving the CTL magnitude and quality. In BALB/c mice with severe combined immune deficiency, primary T cells expressing chimeric receptors that were reactive for HER2 proteins were tested for their adjuvant therapeutic efficacy against mouse mammary carcinoma 4T1.2 cells expressing human erbB-2 in comparison with the effects of the commonly used adjuvants, 5-FU, as well as doxorubicin and Herceptin [18]. In this study, adjuvant therapy using T cells significantly improved the survival rates of mice when compared with mice treated with either one of these drugs. It seems likely that these animal models might be useful for designing optimal protocols for immune-based therapies that are best suited for clinical trials against breast cancer and HER2-positive malignancies.

In this study, we observed that when animals were challenged with CT26/HER2 vs. 4T1.2/HER2 tumor cells, CT26/HER2 cells formed tumors that continued to grow, while 4T1.2/HER2 cells formed tumors that eventually regressed. Contrary to the behavior of CT26/HER2 cells, 4T1.2/HER2 cells induced HER2_63–71_-specific CD8+ CTL responses, resulting in tumor regression. However, CT26/HER2 cells induced higher levels of IFN-γ production in an antigen-non-specific manner and expressed CD80 on their cell surface, unlike 4T1.2/HER2 cells. The tumor tissues of CT26/HER2 tumor-bearing mice also had dramatically increased levels of myeloid-derived suppressor cells (MDSCs). However, IFN-γ, CD80 and MDSCs were found to be not associated with tumor progression in the CT26/HER2 model. Overall, these data show that, in contrast to the behavior of CT26/HER2 tumors, 4T1.2/HER2 tumors regress via the induction of HER2_63–71_-specific CD8+ CTL activity in animals and that CD80 is not associated with the regression of this tumor type.

## RESULTS

### CT26/HER2 cells formed tumors that continued to grow, whereas 4T1.2/HER2 cells formed tumors that regressed following the induction of antitumor immunity in CT26/HER2 cells

**Figure 1:**
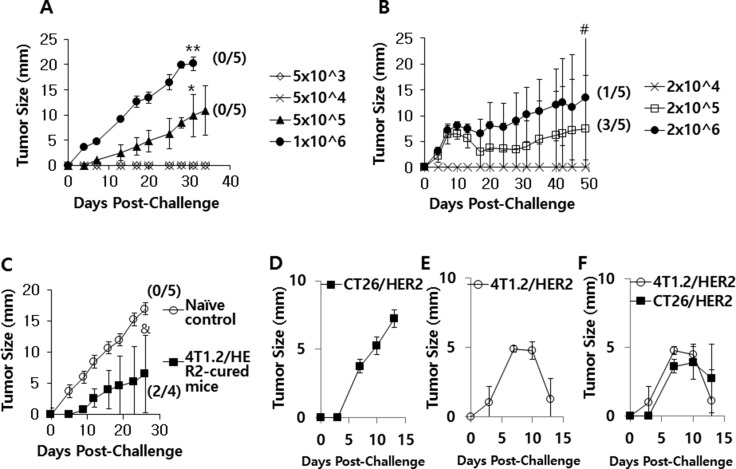
The tumor growth patterns of CT26/HER2 vs. 4T1.2/HER2 cells in mice BALB/c mice (*n* = 5/group) were challenged s.c with 5 × 10^3^ to 1 × 10^6^ CT26/HER2 cells per mouse (**A**) and 2 × 10^4^ to 2 × 10^6^ 4T1.2/HER2 cells per mouse (**B**). (**C**) The 4 tumor (4T1.2/HER2)-cured mice from Figure 1B were re-challenged s.c. with 1 × 10^6^ CT26/HER2 cells per mouse at 50 days post-challenge, along with 5 naïve mice. Tumor-cured animals were denoted as those showing complete tumor eradication at 50 days following tumor cell challenge. (**D**–**F**) BALB/c mice (*n* = 5/group) were challenged s.c with 5 × 10^5^ CT26/HER2 cells per mouse in the right flank (D), 2 × 10^5^ 4T1.2/HER2 cells per mouse in the left flank (E) or both (F). Tumor sizes were measured over time. The values and bars represent tumor sizes and SDs, respectively. The numbers in (/) denote the number of mice without tumors/the number of mice tested. *Statistically significant compared with 5 × 10^4^ cells per mouse. **Statistically significant compared with 5 × 10^5^ cells per mouse. ^#^Statistically significant compared with 2 × 10^4^ cells per mouse. ^&^Statistically significant compared with naïve control.

When mice were challenged with an increasing dose of CT26/HER2 cells (5 × 10^3^, 5 × 10^4^, 5 × 10^5^ and 1 × 10^6^ cells per mouse), they exhibited a tumor growth pattern that occurred in a tumor cell challenge dose-dependent manner (Figure 1A). In contrast, 4T1.2/HER2 cells formed tumors in mice that subsequently regressed (Figure 1B). In particular, 3 of the 5 mice that had been challenged with 2 × 10^5^ 4T1.2/HER2 cells per mouse showed complete tumor regression, while 1 of the 5 mice that had been challenged with 2 × 10^6^ 4T1.2/HER2 cells per mouse showed complete tumor regression. As tumor regression was not detectable in the CT26/HER2 cell-challenged mice (Figure 1A), we speculated that CT26/HER2 cells might possess the capacity to resist the antitumor immunity that was induced by the CT26/HER2 cells. To test this possibility, we challenged the four 4T1.2/HER2 tumor-cured animals from Figure 1B with 1 × 10^6^ CT26/HER2 cells per mouse and measured tumor growth. As seen in Figure 1C, the CT26/HER2 cells grew significantly less in the 4T1.2/HER2 tumor-cured mice over the measured time points than in the age-matched control mice. In this study in particular, 2 of the 4 mice that had been challenged with CT26/HER2 cells failed to form a tumor, while all control mice formed tumors. These results suggest that CT26/HER2 cells are still susceptible to the antitumor immunity induced by 4T1.2/HER2 cells but that they are unable to induce antitumor immunity on their own. We also checked the reciprocal relationship between CT26/HER2 and 4T1.2/HER2 tumors by challenging mice with CT26/HER2 cells on the right flank and 4T1.2/HER2 cells on the left flank. As seen in Figure 1D–1F, the CT26/HER2 tumor formation had no impact on the 4T1.2/HER2 tumor regression, suggesting that antitumor immunity could be induced by 4T1.2/HER2 cells even in a CT26/HER2 tumor-derived *in vivo* environment. Moreover, the growth of established CT26/HER2 tumors was inhibited by 4T1.2/HER2 tumor cells, which corroborated the previous finding that CT26/HER2 cells were susceptible to the antitumor immunity induced by 4T1.2/HER2 cells. Taken together, these results indicate that 4T1.2/HER2 cells can induce antitumor immunity-mediated tumor regression and that CT26/HER2 cells are unable to induce antitumor immunity but remain sensitive to the antitumor immunity induced by 4T1.2/HER2 cells.

### CT26/HER2 and 4T1.2/HER2 cells induced Ag-specific IgG responses, while 4T1.2/HER2 cells induced a Th1 IgG type response

**Figure 2:**
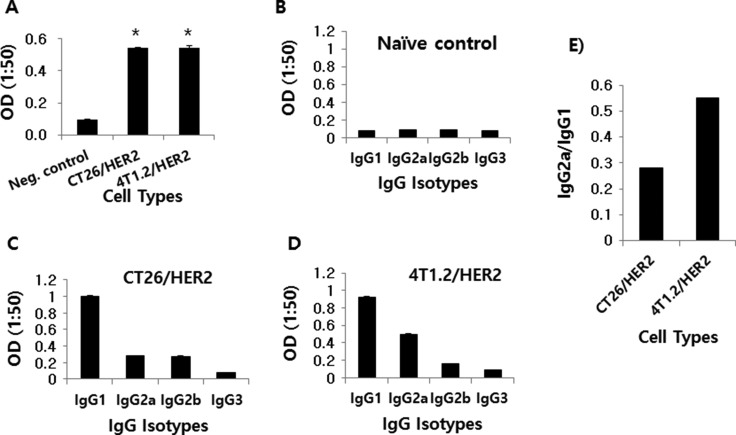
Ag-specific IgG and IgG isotype production in mice challenged with CT26/HER2 vs. 4T1.2/HER2 cells *in vivo* BALB/c mice (*n* = 5/group) were challenged s.c with 5 × 10^5^ CT26/HER2 cells and 2 × 10^5^ 4T1.2/HER2 cells. The animals were bled at 20 days post-challenge. Equally pooled sera were reacted with recombinant HER2 protein to measure total IgG (**A**) and IgG isotypes (**B**–**D**) in ELISA. (**E**) shows the ratio of IgG2a to IgG1. *Statistically significant compared with negative control.

We next tested whether CT26/HER2 and 4T1.2/HER2 cells might induce HER2-specific IgG responses in animals. As shown in Figure 2A, both the CT26/HER2 and 4T1.2/HER2 cells dramatically elicited Ag-specific IgG responses when they were injected into animals. When Ag-specific IgG isotypes were measured, animals that had been challenged with 4T1.2/HER2 cells produced more IgG2a than IgG1 relative to the levels produced in the CT26/HER2 cell-challenged mice (Figure 2B–2E). These results show that 4T1.2/HER2 cells could induce Ag-specific IgG isotype responses towards a more Th1 type than CT26/HER2 cells.

### Immune cells from 4T1.2/HER2 tumor-bearing and tumor-regressed mice produced IFN-γ in response to HER2 class I peptides, while those from CT26/HER2 tumor-bearing mice produced extremely high levels of IFN-γ independent of HER2 class I peptide stimulation

**Figure 3:**
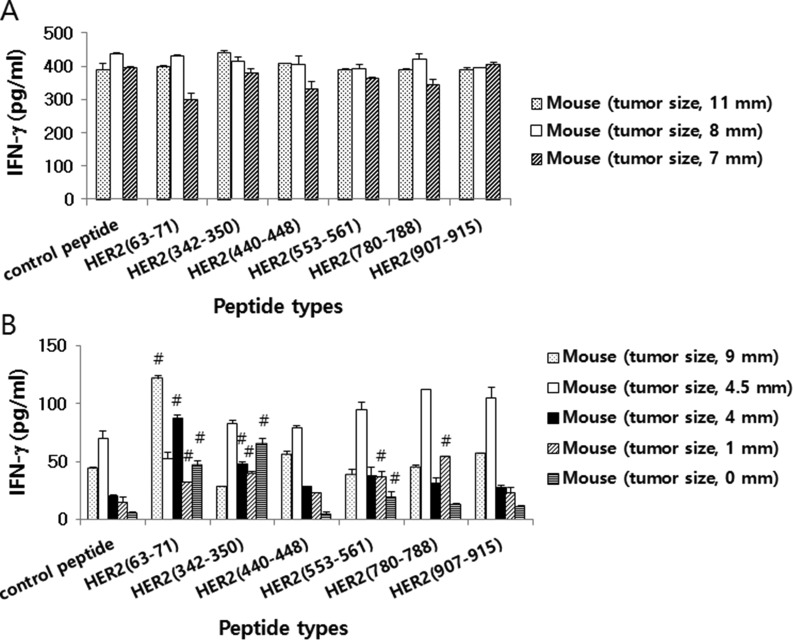
HER2 class I peptide-specific IFN-γ production levels of mice challenged with CT26/HER2 vs. 4T1.2/HER2 cells BALB/c mice (*n* = 5/group) were challenged s.c. with 5 × 10^5^ CT26/HER2 cells (**A**) and 2 × 10^5^ 4T1.2/HER2 cells (**B**). The animals formed tumors that continued to grow or regress. At 13 days following tumor cell challenge, the mice were checked for their tumor sizes and then sacrificed to obtain splenocytes. The splenocytes were isolated and stimulated *in vitro* with different HER2 class I peptides or control E7 peptides. After 2 days of stimulation, the cell supernatants were collected and measured for IFN-γ levels. The values and bars represent IFN-γ levels and SDs, respectively. ^#^indicates a more than two-fold increase in IFN-γ production compared with control peptides.

We next tested whether tumor cells might be able to induce Ag-specific cellular responses in animals. For this test, we evaluated the ability of immune cells isolated from tumor (CT26/HER2 vs. 4T1.2/HER2)-bearing mice to produce IFN-γ in response to the HER2 class I peptides, HER2_63–71_, HER2_342–350_, HER2_440–448_, HER2_553–561_, HER2_780–788_ and HER2_907–915_. It has been reported that HER2 peptides (HER2_63–71_, HER2_342–350_, HER2_440–448_, HER2_553–561_, HER2_780–788_, HER2_907–915_) have a high binding affinity for MHC class I (H-2K^d^) and are potentially recognized by immune cells [19, 20]. The data from Figure 3A show that immune cells from CT26/HER2 tumor-bearing mice produced an extremely high level of IFN-γ in a manner that was independent of HER2 class I peptide stimulation. In contrast, immune cells from 4T1.2/HER2 tumor-bearing and tumor-cured mice produced IFN-γ in a somewhat HER2 peptide-dependent manner (Figure 3B). Specifically, immune cells from 4 of the 5 tumor-bearing and tumor-cured mice showed a more than 2-fold increase in IFN-γ levels in response to HER2_63–71_ compared with the response to the control peptides. Additionally, immune cells from 3 of the 5 tumor-bearing and tumor-cured mice showed a more than 2-fold increase in IFN-γ production in response to HER2_342–350_ compared with the response to the control peptides. Moreover, immune cells from 2 of the 5 tumor-bearing and tumor-cured mice showed a more than 2-fold increase in IFN-γ production in response to HER2_553–561_ compared with the response to the control peptides. Immune cells from 1 of the 5 tumor-bearing and tumor-cured mice showed a more than 2-fold increase in IFN-γ production in response to HER2_780–788_ compared with the response to the control peptides, and none of the immune cells from the tumor-bearing and tumor-cured mice showed a more than 2-fold change in IFN-γ production in response to HER2_440–448_ or HER2_907–915_ compared with the response to the control peptides. Moreover, immune cells from mice bearing no or smaller tumors (mean tumor sizes; 0, 1 and 4 mm) produced dramatically higher levels of IFN-γ following stimulation with the HER2 class I peptides HER2_63–71_ and HER2_342–350_ compared with the response to the control peptides.

### HER2_63-71_-specific CD8+ CTL lytic responses were responsible for tumor regression in 4T1.2/HER2 tumor-bearing mice

**Figure 4:**
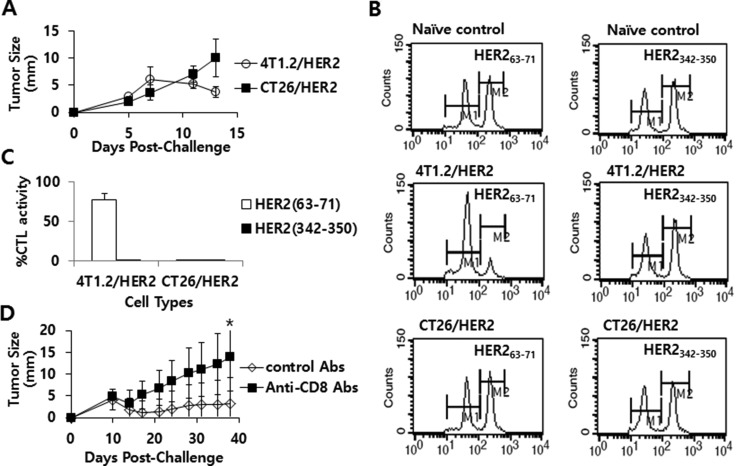
The tumor growth patterns of CT26/HER2 vs. 4T1.2/HER2 cells over time, the Ag-specific CTL lytic activity *in vivo* and the effect of CD8+ T cell depletion on tumor regression in mice challenged with 4T1.2/HER2 cells BALB/c mice (*n* = 5/group) were challenged s.c with 5 × 10^5^ CT26/HER2 cells per mouse and 2 × 10^5^ 4T1.2/HER2 cells per mouse. Tumor sizes were measured over time (**A**). The mice were tested for *in vivo* CTL lytic activity for 48 h at 11 days post-challenge, as described in the Methods and Materials (**B**). (**C**) shows %CTL activity in response to HER2_63-71_ and HER2_342-350_. The values and bars represent tumor sizes or %CTL activity, and SDs, respectively. (**D**) BALB/c mice (*n* = 5/group) were challenged with 2 × 10^5^ 4T1.2/HER2 cells per mouse and then injected with 100 μg of control or anti-CD8 Ab at 9, 12 and 15 days post-challenge. Tumor sizes were measured over time. The values and bars represent tumor sizes and SDs, respectively. *Statistically significant compared to control Abs.

As we previously observed that immune cells from at least 3 of the 5 4T1.2/HER2 tumor-bearing and tumor-cured mice showed a more than 2-fold increase in IFN-γ production in response to the HER2 peptides HER2_63–71_ and HER2_342–350_ relative to the response to the control peptides, we next examined whether 4T1.2/HER2 tumor-bearing mice might have CTL lytic responses to these two peptides. Figure 4A shows the tumor growth patterns of 4T1.2/HER2 and CT26/HER2 cells over time following a tumor cell challenge. Consistent with our previous observations (Figure 1), 4T1.2/HER2 cells formed tumors that eventually regressed, while CT26/HER2 cells formed tumors that continued to grow. Subsequently, 4T1.2/HER2 and CT26/HER2 tumor-bearing mice were tested for *in vivo* CTL lytic activity. As shown in Figure 4B and 4C, 4T1.2/HER2 tumor-bearing mice showed CTL lytic activity in response to HER2_63–71_ peptides but not to HER2_342–350_, while CT26/HER2 tumor-bearing mice displayed no such activity. In particular, 4T1.2/HER2 tumor-bearing mice showed approximately 80% HER2_63–71_-specific CTL lytic activity, whereas CT26/HER2 tumor-bearing mice displayed no HER2_63–71_-specific CTL lytic activity (Figure 4C). We further tested whether the depletion of CD8+ T cells from mice challenged with 4T1.2/HER2 cells might result in the loss of tumor regression following tumor cell challenge. The data from Figure 4D show that the injection of anti-CD8 Abs led to a loss of tumor regression following a tumor cell challenge when compared with the effects of an injection of control Abs, which failed to show such effects. These results, along with our previous observations, support the notion that 4T1.2/HER2 cells can induce HER2_63–71_-specific CD8+ CTL responses that lead to tumor regression. However, in the case of CT26/HER2 cells, a lack of the ability to induce HER2_63–71_-specific CTL responses might have been responsible for the continuous tumor growth observed in mice challenged with these cells.

### Systemic IFN-γ levels were significantly higher in CT26/HER2 tumor-bearing mice than in 4T1.2/HER2 tumor-bearing mice, despite their different tumor and spleen sizes while no CT26/HER2 tumor regression was observed in IFN-γ knockout mice

**Figure 5:**
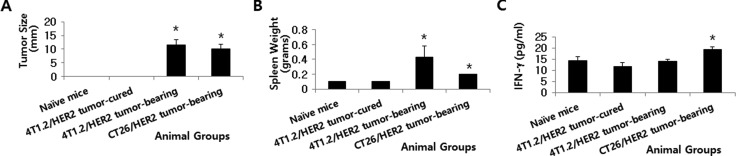
Tumor size, spleen weight and IFN-γ levels in mice with and without 4T1.2/HER2 and CT26/HER2 tumors BALB/c mice were challenged s.c. with 4T1.2/HER2 (2 × 10*5* cells/mouse) and CT26/HER2 cells (5 × 10*5* cells/mouse). (**A**) Tumors (10 mm in size) in the 4T1.2/HER2 and CT26/HER2 cell-challenged mice were detectable at 36 days following 4T1.2/HER2 tumor cell challenge and at 23 days following CT26/HER2 tumor cell challenge. 4T1.2/HER2 tumor-cured mice were denoted as mice whose tumors regressed completely at 23 days following tumor cell challenge. These mice were sacrificed in order to measure their spleen weights (**B**). (**C**) BALB/c mice were challenged as previously described. When their tumor sizes had a mean diameter of approximately 10 mm, blood serum samples were collected. Serum samples from 4T1.2/HER2 tumor-cured mice were also collected for this assay. The samples were used to measure IFN-γ levels with an ELISA. The values and bars represent tumor sizes, spleen weights or IFN-γ levels, and SDs, respectively. *Statistically significant compared with naïve mice.

Next, tumor and spleen sizes and systemic IFN-γ levels were compared in CT26/HER2 tumor-bearing vs. 4T1.2/HER2 tumor-bearing mice. We performed this analysis when the CT26/HER2 and 4T1.2/HER2 tumors reached approximately the same sizes. Figure 5A shows the two groups of animals bearing tumors (mean tumor size of approximately 10 mm) when the mice were bled and sacrificed for measurements of spleen size. We also selected one group of 4T1.2/HER2 cell-challenged mice that displayed complete tumor regression in another set of studies. Figure 5B shows the animals’ spleen sizes. 4T1.2/HER2 tumor-cured mice had the same spleen sizes as naive controls. In contrast, 4T1.2/HER2 and CT26/HER2 tumor-bearing mice had spleens that were significantly larger than those of naive controls and 4T1.2/HER2 tumor-cured mice. However, systemic IFN-γ levels were significantly greater in the CT26/HER2 tumor-bearing mice than in the naive controls, 4T1.2/HER2 tumor-cured, and 4T1.2/HER2 tumor-bearing mice, which displayed similar IFN-γ levels (Figure 5C). These results, along with our previous *in vitro* IFN-γ data (Figure 3A), indicate that a high level of systemic IFN-γ might be negatively associated with the induction of antitumor CTL responses in CT26/HER2 tumor-bearing mice.

### CT26/HER2 cells alone expressed CD80 on their cell surface

**Figure 6:**
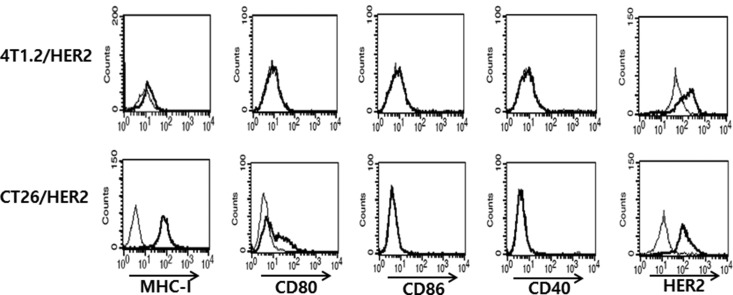
MHC-I, CD80, CD86, CD40 and HER2 antigen expression levels on the surface of 4T1.2/HER2 vs. CT26/HER2 cells. Five × 10^5^ tumor cells were reacted with FITC-labeled anti-MHC-I (thick line) and isotype control (thin line) Abs and with PE-labeled anti-CD80, anti-CD86, anti-CD40 Abs (thick line) and isotype control Abs (thin line) For HER2 expression, the cells were treated with anti-HER2 mouse serum (thick line) or control serum (thin line), followed by treatment with FITC-labeled anti-mouse IgG.

It is known that during interactions between the antigen-MHC complexes of antigen presenting cells (APCs) and the T cell receptors of T cells, co-stimulatory molecules (CD80 and CD86) can recognize CD28/CTLA-4 on T cells and drive T cells into the cell cycle, leading to T cell activation, differentiation and cytokine production (reviewed in [21, 22]). CD40 is also expressed on APCs, including B cells, dendritic cells (DCs) and monocytes [23, 24] and the interaction between CD40 on APCs and CD40 ligands on T cells leads to the activation of both APCs and T cells [25–27]. In this context, it is possible that CT26/HER2 cells might express antigen-MHC complexes and co-stimulatory molecules on their surface and recognize and activate T cells, leading to higher IFN-γ production. To test this possibility, we measured the expression levels of certain co-stimulatory molecules (CD80, CD86 and CD40) on CT26/HER2 and 4T1.2/HER2 cells. Simultaneously, we measured the expression levels of HER2 and MHC class I antigens on tumor cells. Figure 6 shows that only the CT26/HER2 cells expressed CD80, but they did not express CD86 or CD40. On the other hand, both tumor cells expressed HER2 antigens and MHC class I molecules. Thus, these *in vitro* data suggest that the co-stimulatory molecule, CD80, which was expressed on CT26/HER2 cells, might play a role in producing higher IFN-γ levels by co-stimulating T cells, likely resulting in the suppression of CTL induction during tumor progression.

### CD80 was not associated with tumor growth or regression in CT26/HER2 or 4T1.2/HER2 models

**Figure 7:**
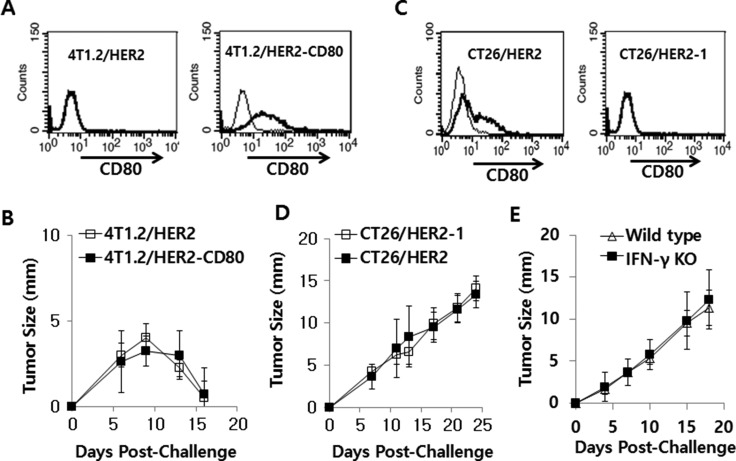
4T1.2/HER2 and CT26/HER2 cell tumor growth patterns with/without CD80 expression and CT26/HER2 cell tumor growth patterns in IFN-γ deficient mice (**A**) 4T1.2/HER2 cells were stably transfected with CD80 cDNA and then selected against hygromycin B. The selected cells tested positive for CD80 based on a FACS analysis. Thin line, control Ab; bold line, anti-CD80 Ab. (**B**) BALB/c mice (*n* = 5/group) were challenged s.c. with wild type or CD80-expressing 4T1.2/HER2 cells (2 × 10^5^ cells/mouse). Tumor sizes were measured over time. (**C**) CD80 expression levels on wild type CT26/HER2 and CT26/HER2-1 cells, as determined via FACS analysis. Thin line, control Ab; bold line, anti-CD80 Ab. (**D**) BALB/c mice (*n* = 5/group) were challenged s.c. with CT26/HER2 and CT26/HER2-1 cells (5 × 10^5^ cells/mouse). (**E**) BALB/c IFN-γ knockout and wild type mice (*n* = 5/group) were challenged s.c. with CT26/HER2 cells (5 × 10^5^ cells/mouse). Tumor sizes were measured over time. The values and bars represent tumor sizes and SDs, respectively.

To investigate whether CD80 might be negatively associated with tumor regression, we transfected 4T1.2/HER2 cells with pCEP4-CD80 and selected a tumor cell clone expressing CD80 for use in a tumor cell challenge study. As seen in Figure 7A, 4T1.2/HER2 cells transfected with pCEP4-CD80 expressed CD80 on the cell surface, which did not occur in wild type 4T1.2/HER2 cells. When CD80-expressing and CD80-non-expressing 4T1.2/HER2 cells were injected into animals, they formed tumors that subsequently regressed in a similar fashion (Figure 7B). This result suggests that CD80 has no direct role in tumor regression in the 4T1.2/HER2 tumor model. Previously, we selected CT26/HER2-1 cells that did not express CD80 on their surface [28], as shown in Figure 7C. We challenged mice with CT26/HER2 and CT26/HER2-1 cells and measured the sizes of the tumors. As shown in Figure 7D, there was no difference in the tumor growth patterns between the 2 tumor cell types, suggesting that CD80 is not associated with tumor progression in the CT26/HER2 tumor model. Therefore, these *in vivo* data show that CD80 is not associated with tumor progression or regression in the two tumor models (4T1.2/HER2 and CT26/HER2). Moreover, when IFN-γ knockout mice were challenged with CT26/HER2 tumor cells, they displayed tumor growth in a manner similar to wild type mice (Figure 7E). Therefore, this result indicates that IFN-γ is also not associated with tumor progression in the CT26/HER2 tumor model.

### IFN-γ and co-stimulatory molecule levels in the TC-1 and MC32 tumor models

**Figure 8:**
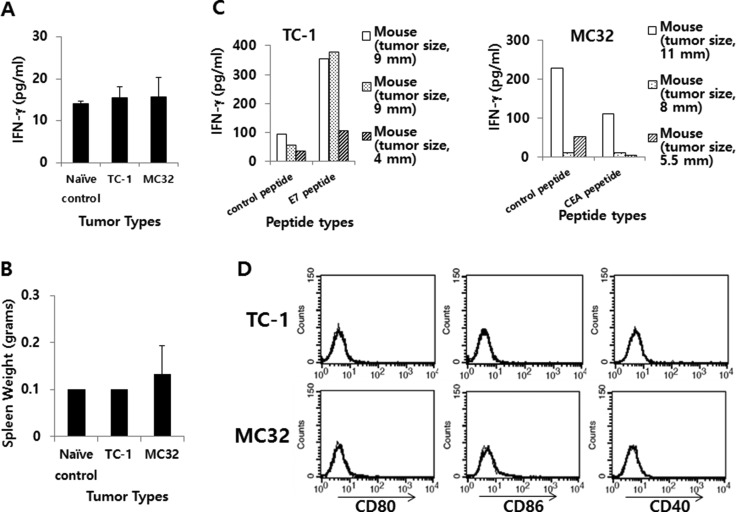
IFN-γ and the expression level of co-stimulatory molecules in TC-1 and MC32 tumor models (**A**) Each group of C57BL/6 mice (*n* = 3/group) was challenged s.c. with TC-1 and MC32 cells (5 × 10^5^ cells/mouse). When tumor sizes reached 7–8 mm in mean diameter (9, 9 and 4 mm for TC-1 tumor; 11, 8 and 5,5 mm for MC32 tumor), the mice were bled and sera were collected to measure systemic IFN-γ levels using ELISA. (**B**) The mice were sacrificed to measure their spleen weights. (**C**) The splenocytes were isolated and reacted for 2 days with class I CTL peptides (control CEA vs. E7 peptides for TC-1 tumor; control E7 vs. CEA peptides for MC32 tumor). The collected cell supernatants were used to measure IFN-γ levels using ELISA. (**D**) Five × 10^5^ TC-1 and MC32 tumor cells were treated with PE-labeled anti-CD80, anti-CD86 and anti-CD40 (thick line), as well as control Abs (thin line) to measure the expression of CD80, CD86 and CD40 molecules using a flow cytometer. The values and bars show IFN-γ levels or spleen weights, and SDs, respectively.

We next examined the relationship between IFN-γ levels and the expression status of co-stimulatory molecules in 2 other tumor models (TC-1 and MC32 models). We previously reported that these 2 tumor cells were able to form tumors that continued to grow in mice [29–32] in a manner similar to that of CT26/HER2 cells, as we observed here. For this test, we challenged mice with TC-1 and MC32 tumor cells and collected blood samples when the tumors reached 7–8 mm in mean diameter. The serum samples were used to measure systemic IFN-γ levels. Simultaneously, the mice were sacrificed to obtain splenocytes, which were subsequently incubated with class I CTL peptides to measure *in vitro* IFN-g production levels. As shown in Figure 8A, the TC-1 and MC32 tumor-bearing mice had systemic IFN-γ levels that were similar to those of the naive control mice. These mice showed an insignificant degree of splenomegaly (Figure 8B). Furthermore, all of the immune cells from the TC-1 and MC32 tumor-bearing mice produced IFN-γ in somewhat antigen-specific and non-specific manners, depending on the tumor cell type (Figure 8C), suggesting that the IFN-γ levels might have no direct association with tumor progression. We also measured the expression levels of co-stimulatory molecules on the surface of TC-1 and MC32 cells. As seen in Figure 8D, the TC-1 and MC32 cells displayed no CD80, CD86 or CD40 surface expression, supporting the notion that CD80 is not directly associated with IFN-γ production and tumor progression.

### Evaluation of immune cell types in the tumor-draining lymph nodes (TDLNs) and tumor from CT26/HER2 tumor-bearing and 4T1.2/HER2 tumor-bearing mice

**Figure 9:**
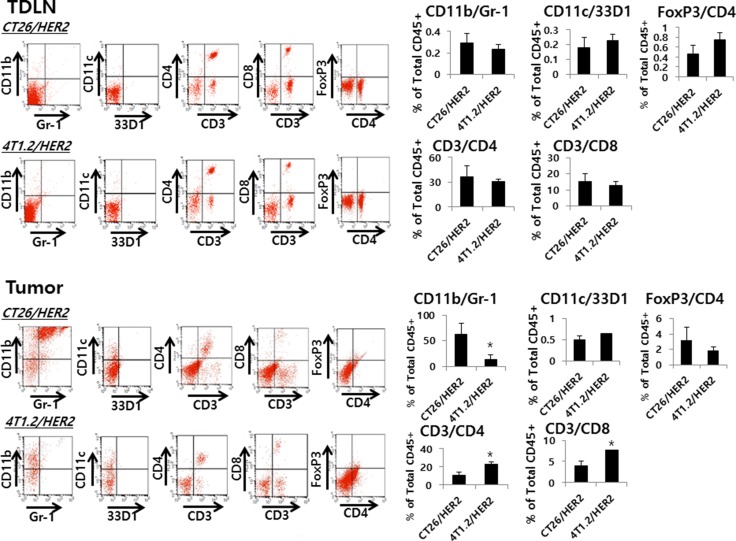
Myeloid and lymphoid cell populations in TDLNs and tumors from CT26/HER2 and 4T1.2/HER2 tumor-bearing mice BALB/c mice (*n* = 5/group) were challenged with 5 × 10^5^ CT26/HER2 cells per mouse and 2 × 10^5^ 4T1.2/HER2 cells per mouse as in Figure 4A. At 12 days post-challenge, the mice were sacrificed and tested for the percentage of myeloid cells (CD11b+/Gr-1+ and CD11C+/33D1+ cells) and lymphoid cells (CD3+/CD4+, CD3+/CD8+ and CD4+/FoxP3+ cells) among anti-CD45 Ab-gated cells in the TDLNs and tumors of each mouse, as described in the Methods and Material. The values and bars represent mean cell percentages among total CD45+ cells and SDs, respectively. *Statistically significant compared with CT26/HER2.

TDLNs and tumor tissues of CT26/HER2 tumor-bearing and 4T1.2/HER2 tumor-bearing mice were analyzed for changes in the immune cell subsets by flow cytometry. As seen in Figure 9, there was no significant difference between CT26/HER2 tumor-bearing and 4T1.2/HER2 tumor-bearing mice with respect to the percentages of CD11b+/Gr-1+ cells, CD11c+/33D1+ cells, CD3+/CD4+ T cells, CD3+/CD8+ T cells and CD4+/FoxP3+ Treg cells in the TDLNs. However, the tumor of CT26/HER2 tumor-bearing mice had dramatically increased levels of CD11b+/Gr-1+ cells than that of 4T1.2/HER2 tumor-bearing mice. On the other hand, the tumor of 4T1.2/HER2 tumor-bearing mice had dramatically increased levels of CD3+/CD4+ and CD3+/CD8+ T cells than that of CT26/HER2 tumor-bearing mice. Thus, these results suggest that the presence of MDSCs (expressing a CD11b+/Gr-1+ phenotype) in the tumor tissues might be responsible for inhibiting Ag-specific CTL induction and/or blocking antitumor CTL effector functions, allowing for continuous tumor growth in the CT26/HER2 tumor model.

### Gemcitabine inhibited tumor growth without any effects on Ag-specific IFN-γ and CD8+ CTL induction, as well as NK cell induction in the CT26/HER2 tumor model

**Figure 10:**
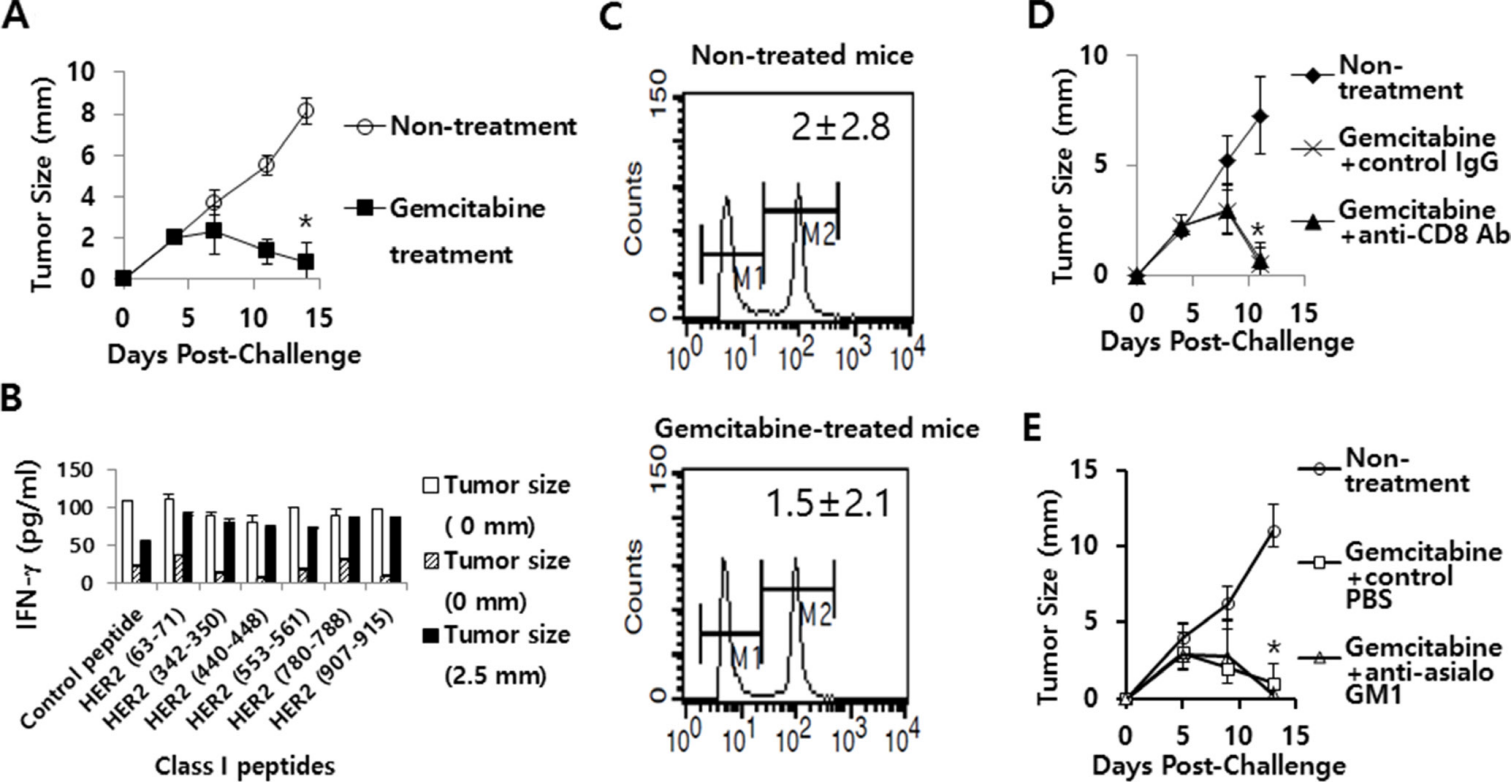
The effects of gemcitabine on tumor growth, IFN-γ induction and CTL lytic activity in responses to HER2 class I peptides, as well as the role of CD8+ T and NK cells in gemcitabine-induced tumor regression (**A**) BALB/c mice (*n* = 6/group) were challenged s.c. with 5 × 10^5^ CT26/HER2 cells per mouse. The mice were injected twice a week for 2 weeks starting from day 5 of post-tumor challenge with gemcitabine. Tumor sizes were measured over time. The values and bars represent mean tumor sizes and SDs, respectively. (**B**) At 14 days following tumor cell challenge (from Figure 10A), the 3 tumor-regressed mice were sacrificed to obtain spleen. Splenocytes were isolated and stimulated *in vitro* with different HER2 class I peptides or control E7 peptides. After 2 days of stimulation, the cell supernatants were collected and measured for IFN-γ levels. The values and bars represent IFN-γ levels and SDs, respectively. (**C**) At 14 days following tumor cell challenge (from Figure 10A), the 3 remaining tumor-regressed mice receiving gemcitabine treatment, as well as the 3 un-treated control tumor-bearing mice were tested for *in vivo* CTL lytic activity in response to HER2_63-71_. (**D**, **E**) BALB/c mice (*n* = 5/group) were challenged s.c. with 5 × 10^5^ CT26/HER2 cells per mouse. The mice were injected twice a week for 2 weeks starting from day 5 of tumor challenge with gemcitabine. Animals were also injected i.p. with anti-CD8 (D) and control Abs at 2, 5 and 8 days post-challenge, and anti-NK (E) and control PBS at 2, 5 and 10 days post-challenge. Tumor sizes were measured over time. The values and bars represent mean tumor sizes and SDs, respectively. *Statistically significant compared to non-treatment.

Gemcitabine and anti-Gr-1 Ab treatment has been reported to deplete MDSCs in a C26 tumor model [33]. To test whether gemcitabine might induce antitumor activity by deleting MDSCs, we challenged mice with CT26/HER2 tumor cells, followed by treatment with gemcitabine. As seen in Figure 10A, gemcitabine treatment displayed a marked and significant level of tumor regression over the time points compared with non-treatment. In this context, it was hypothesized that gemcitabine might induce tumor regression through the induction of Ag-specific IFN-γ and CTL responses in this tumor model. To test this possibility, we performed an IFN-γ release assay. When immune cells from the tumor-regressed mice following treatment with gemcitabine were stimulated *in vitro* with HER2 class I peptides, they failed to produce IFN-γ to a significant level in response to HER2 class I peptides compared with control peptides (Figure 10B). These mice also failed to display *in vivo* CTL lytic activity in response to HER2_63–71_ (Figure 10C). Thus, these results suggest that gemcitabine-induced tumor regression is not immune-mediated. To further confirm this finding, CD8+ T and NK cell depletion assays were performed. As shown in Figure 10D and 10E, the tumor-bearing mice receiving either gemcitabine plus anti-CD8 Abs (D) or gemcitabine plus anti-NK Abs (E) displayed tumor regression similar to those receiving gemcitabine and control IgG/phosphate-buffered saline (PBS). Taken together, these results indicate that the antitumor effect of gemcitabine is not mediated by antitumor immunity but possibly by its direct cytotoxic activity on tumor cells in this model.

### Effects of gemcitabine and anti-Gr-1 Abs on tumor growth and MDSC levels in the CT26/HER2 tumor model

**Figure 11:**
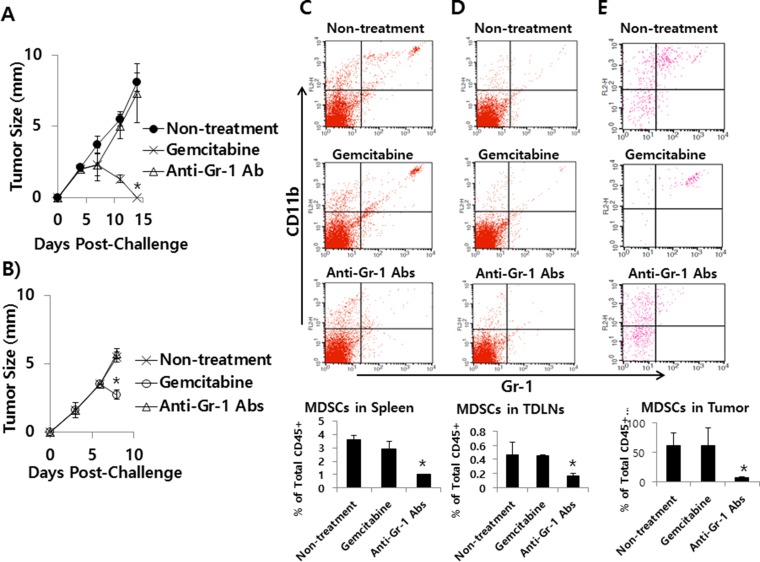
The effects of gemcitabine and anti-Gr-1 Ab treatment on tumor growth and MDSC levels in the spleen, TDLN and tumor (**A**) BALB/c mice (*n* = 5/group) were challenged s.c. with 5 × 10^5^ CT26/HER2 cells per mouse. The mice were injected twice a week for 2 weeks starting from day 5 of post-tumor challenge with gemcitabine. The mice were also injected with anti-Gr-1 Abs at 4 and 11 days post-challenge. Tumor sizes were measured over time. The values and bars represent mean tumor sizes and SDs, respectively. (**B**–**E**) BALB/c mice (*n* = 5/group) were challenged s.c. with 5 × 10^5^ CT26/HER2 cells per mouse. When tumor sizes were approximately 3.5 mm at 6 days post-challenge, the mice were injected with gemcitabine and anti-Gr-1 Abs. Tumor sizes were measured at 8 days post-challenge (B) and the mice were subsequently sacrificed to measure MDSC levels in the spleen (C), TDLN (D) and tumor (E) of each mouse. The values and bars represent mean tumor sizes and MDSC levels, and the SDs, respectively. *Statistically significant compared to non-treatment.

Next, we tested whether anti-Gr-1 Ab treatment might induce tumor regression through depletion of MDSCs in the CT26/HER2 tumor model in parallel with gemcitabine treatment. As seen in Figure 11A, gemcitabine treatment displayed a dramatic level of tumor regression as observed in Figure 10A. However, anti-Gr-1 Ab treatment failed to show such an effect. A similar finding was obtained when tumor (approximately 3.5 mm in mean size)-bearing mice were treated with gemcitabine and anti-Gr-1 Abs (Figure 11B). We further tested whether gemcitabine and anti-Gr-1 Ab treatment might lead to depletion of MDSCs. As seen in Figure 11C–11E, administration of the tumor-bearing mice with anti-Gr-1 Abs led to depletion of MDSCs in the spleen, TDLN and tumor of the tumor-bearing mice. In this case, it is notable that the percentage of MDSCs in the spleen of naïve mice was approximately 3% (data not included). Thus, this result, along with tumor growth data, suggests that MDSCs are not responsible for a lack of tumor regression in the CT26/HER2 model. On the other hand, gemcitabine treatment had no effects on MDSC depletion, supporting the notion that gemcitabine suppresses tumor growth mainly by its direct cytotoxic activity on tumor cells. Taken together, these results show that, although a dramatically increased number of MDSCs are present in the tumor tissue of CT26/HER2 tumor-bearing mice, they may not be involved in promoting tumor growth in this model.

## DISCUSSION

In the current study, we observed that when animals were challenged with 4T1.2/HER2 tumor cells, they were able to induce Ag-specific CD8+ CTL lytic activity, leading to tumor regression. However, CT26/HER2 tumor cells failed to induce such responses but were still susceptible to the Ag-specific CD8+ CTL activity induced by 4T1.2/HER2 cells. Moreover, the presence of CT26/HER2 tumors was unable to prevent 4T1.2/HER2 cells from inducing tumor regression, suggesting that the CT26/HER2 tumor-derived *in vivo* environment may not have any influence on the immunogenicity of 4T1.2/HER2 cells. The results of these collective animal studies show that the HER2 antigens of 4T1.2/HER2 cells might be cross-processed and cross-presented by APCs to naive T cells that recognize HER2 antigens, which does not appear to occur for the HER2 antigens of CT26/HER2 cells. This unique property of 4T1.2/HER2 cells is unexpected as the other tumor cell types, including CT26/HER2 cells, tested in this study did not show any spontaneous tumor regression via the induction of antitumor immunity in the mice. For instance, when mice were challenged with tumor cells in the 2 animal tumor models (TC-1 and MC32 models), tumors formed and continued to grow [29–32]. However, despite this difference in tumor control, both CT26/HER2 and 4T1.2/HER2 tumor cells were able to induce Ag-specific IgG responses in animals, possibly because of the location of HER2 antigen expression on the cell membrane. In particular, IgG2a was induced to a greater extent than IgG1 in 4T1.2/HER2 tumor-bearing mice but not in CT26/HER2 tumor-bearing mice, suggesting that 4T1.2/HER2 cells may be more likely to induce Th1 type immune responses. In our previous tumor models, TC-1 (expressing human papillomavirus 16 E6/E7) cells, in particular, were unable to induce Ag-specific IgG production *in vivo*, while MC32 cells (expressing human carcinoembryonic antigen [CEA]) induced Ag-specific IgG responses (data not shown). This difference appears to result from the cellular location of the tumor antigens (i.e., the nucleus in TC-1 cells vs. the cell membrane-bound and secretory locations in MC32 cells). In our previous observation, moreover, the tumor antigens of TC-1 and MC32 tumor cells are not cross-presented for induction of Ag-specific CTL responses in TC-1 and MC32 models. Taken together, the data from these animal tumor model studies show that the induction status of Ag-specific antibodies and the cellular and tumor regression responses might be determined by the tumor cell type and the location of tumor antigen expression.

We also observed that immune cells from CT26/HER2 tumor-bearing mice produced extremely high levels of IFN-γ in an antigen-non-specific manner. However, immune cells from 4T1.2/HER2 tumor-cured mice and 4T1.2/HER2 tumor-bearing mice with smaller tumors induced a more than 2-fold increase in IFN-γ production mainly in response to 2 of the HER2 class I peptides tested, HER2_63–71_ and HER2_342–350_. These *in vitro* data are not in line with our *in vivo* CTL lytic activity data, which showed that CTL lytic activity in 4T1.2/HER2 tumor-bearing mice was induced by HER2_63–71_ peptides but not by HER2_342–350_ peptides. Presently, it is still unclear why HER2_342–350_–specific CTL lytic activity was not inducible in animals in which immune cells could recognize the HER2_342–350_ peptides and produce IFN-γ. In spite of this conflicting result, our *in vivo* CTL data are compatible with those of other studies, including our previous studies demonstrating that HER2_63–71_-specific CD8+ CTLs are crucial for tumor control [20, 28]. We further speculated that an extremely high level of IFN-γ might be associated with the inhibition of Ag-specific CD8+ CTL induction in the CT26/HER2 tumor model. IFN-γ has long been considered to provide antitumor benefits by inhibiting tumor cell proliferation and/or by augmenting antigen processing for MHC class I and II pathways (reviewed in [34]). On the other hand, IFN-γ is also known to promote tumor cell growth via two main mechanisms: 1) IFN-γ induces a high level of programmed death (PD)-L1 expression on the tumor cell surface [35], which inhibits T cell activation by binding to PD-L1 receptors expressed on T cells, and 2) IFN-γ increases the expression of non-cognate MHC class I molecules on tumor cells [36], which likely reduces the capacity of tumor cells to be recognized by Ag-specific CTLs. In our observations, CT26/HER2 tumor–bearing mice with splenomegaly had significantly higher systemic IFN-γ levels than the other animal groups (4T1.2/HER2 tumor-bearing mice with splenomegaly, 4T1.2/HER2 tumor-cured mice and naive control mice), again suggesting a possible role of IFN-γ in inhibiting CTL induction. Moreover, CT26/HER2 cells, but not 4T1.2/HER2 cells, expressed CD80 on their cell surface. It is known that CD80 molecules can recognize CD28/CTLA-4 on T cells and drive T cells into the cell cycle, inducing T cell activation and cytokine production (reviewed in [21, 22]). Therefore, it is possible that CT26/HER2 cells expressing CD80 may act as APCs, activating T cells and causing them to produce higher levels of IFN-γ, which likely block CTL induction during tumor progression. However, this was demonstrated to be unlikely, as we found that CD80-transfected 4T1.2/HER2 cells behaved similarly to wild type 4T1.2/HER2 cells (without CD80) in terms of tumor regression. These data are also in line with the data from the CT26/HER2 model showing that CT26/HER2 cells not expressing CD80 behaved in a similar manner to wild type CT26/HER2 cells in terms of tumor progression. Therefore, CD80 does not appear to be associated with tumor progression through an increase in the production of IFN-γ in the CT26/HER2 tumor model. These data are also compatible with our data obtained from other tumor cells (TC-1 and MC32 cells), which did not express CD80, CD86 or CD40 and did not show an increase in IFN-γ levels but still displayed tumor progression. For instance, even in the absence of increased systemic IFN-γ levels, TC-1 and MC32 cells managed to form tumors, as previously observed [32, 37], supporting the notion that IFN-γ might not have an inhibitory role in inducing Ag-specific CD8+ CTL responses. This is further confirmed by our IFN-γ deficient animal study showing no difference in the growth patterns of CT26/HER2 tumor cells between wild type and IFN-γ knockout mice. Taken together, these data suggest that CD80 molecules are not associated with tumor progression through the induction of higher IFN-γ levels in the CT26/HER2 tumor model. Moreover, a causal relationship between increased IFN-γ levels and the lack of CTL induction is unlikely in this tumor model.

In this study, 4T1.2/HER2 tumor-bearing mice also showed tumor regression even in the presence of CT26/HER2 tumor formation, which occurred in a manner similar to tumor regression in the absence of CT26/HER2 tumor formation. These results suggest that the CT26/HER2 tumor-derived *in vivo* environment, including higher levels of IFN-γ, might not have any inhibitory effect on the induction of Ag-specific CTLs in response to 4T1.2/HER2 cells. Furthermore, this finding rules out the possibility that the changes in IFN-γ levels engendered by CT26/HER2 cells might increase the expression of non-cognate MHC class I molecules on tumor cells, thus leading to the inhibition of tumor recognition by Ag-specific CTLs. This phenomenon occurred because the *in vivo* CT26/HER2 cell environment, which included higher IFN-γ levels, was not effective at blocking 4T1.2/HER2 tumor regression. We also observed that CT26/HER2 cells expressed PD-L1 on their surface [28]. In that study, treatment of CT26/HER2 tumor-bearing mice with anti-PD-L1 antibodies failed to show any effects on Ag-specific CTL induction and tumor growth inhibition, suggesting that PD-L1 is not associated with the inhibition of Ag-specific CTL induction in the CT26/HER2 model. Moreover, there was no significant difference between CT26/HER2 tumor-bearing and 4T1.2/HER2 tumor-bearing mice with respect to the populations of MDSCs, DCs and Treg cells in the TDLNs, suggesting that MDSCs, DCs and Treg cells may not be associated with the regulation of Ag-specific CTL induction in CT26/HER2 tumor-bearing mice. Taken together, it is still unclear why the HER2 antigens of CT26/HER2 cells cannot be cross-processed and presented to T cells for the induction of Ag-specific CTLs, which does appear to occur for the antigens of 4T1.2/HER2 cells.

In this study, we observed that the tumors of CT26/HER2 tumor-bearing mice had dramatically increased levels of MDSCs than those of 4T1.2/HER2 tumor-bearing mice. It is known that MDSCs function in the induction of antitumor T cell unresponsiveness through various mechanisms, including the generation of immune suppressive cytokines, the production of nitric oxide and reactive oxygen species and the removal of cystine and cysteine from tumor microenvironment [38–40]. However, depletion of MDSCs from tumor-bearing mice by anti-Gr-1 Ab treatment failed to show tumor regression in the CT26/HER2 model, suggesting that MDSCs may not have the ability to block the induction of tumor regression in this model. We also observed that there was no significant difference in the populations of NK and NKT cells between CT26/HER2 and 4T1.2/HER2 tumors (data not included), indicating that NK and NKT cells are not associated with tumor growth regulation. It is known that immune inhibitory molecules, such as TGF-β, indoleamine 2,3-dioxygenase and adenosine are associated with tumor immune evasion (reviewed in [41]). In this context, it is possible that these molecules might be associated with a lack of tumor regression in the CT26/HER2 tumor model. However, this needs to be further clarified. In addition, we also observed in the CT26/HER2 model that gemcitabine treatment displayed a dramatic level of antitumor therapeutic effects even without any effects on MDSC depletion as well as CTL and NK cell activation. It is likely that gemcitabine inhibits tumor growth by its direct cytotoxic effects on tumor cells in this model.

In conclusion, we observed that when animals were challenged with CT26/HER2 and 4T1.2/HER2 cells, the CT26/HER2 cells formed tumors that continued to grow, whereas the 4T1.2/HER2 cells formed tumors that eventually regressed. We found that contrary to the response to the CT26/HER2 cells, the 4T1.2/HER2 cells induced HER2_63–71_-specific CD8+ CTL responses, leading to tumor regression. In contrast, CT26/HER2 cells induced a higher level of IFN-γ production in an antigen-non-specific manner, attracted higher levels of MDSCs in their tumor tissue and expressed CD80 onto their surface, which was not the case for 4T1.2/HER2 cells. However, IFN-γ and MDSCs were found to be not associated with tumor progression in the CT26/HER2 model. Moreover, the introduction of CD80 onto 4T1.2/HER2 cells, as well as the removal of CD80 from CT26/HER2 cells, did not alter their original tumorigenicity. Overall, these data showed that, unlike CT26/HER2 cells, 4T1.2/HER2 cells induced HER2_63–71_-specific CD8+ CTLs, leading to tumor regression, and that CD80 molecules were not associated with this tumor regression.

## MATERIALS AND METHODS

### Animals and cells

Six week-old female BALB/c and C57BL6 mice were purchased from Daehan Biolink (Chungbuk, Korea). BALB/c IFN-γ knockout mice were kindly provided by Y.S. Gho (POSTECH, Korea). The mice were cared for under the guidelines of Kangwon Institutional Animal Care and Use Committee-approved protocol. HER2-expressing CT26 cells (CT26/HER2 cells), a colon cancer cell line originating from BALB/c mice [42], were kindly provided by H.J. Hong (Kangwon National University, Korea). 4T1.2 cells expressing human erbB-2 (4T1.2/HER2 cells), a breast cancer cell line also originating from BALB/c mice, were kindly provided by P. K. Darcy (Peter MacCallum Cancer Centre, East Melbourne, Victoria, Australia). TC-1 and MC32 cells originate from C57BL/6 mice and have been previously tested [29, 32, 43, 44]. The tumor cells were maintained in cDMEM medium (supplemented with 10% fetal bovine serum [FBS], 1% L-glutamine and 1% penicillin/streptomycin), except for the TC-1 cells, which were grown in cRPMI medium (supplemented with 10% FBS, 1% L-glutamine and 1% penicillin/streptomycin) containing 400 μg/ml of G418.

### Tumor cell challenge studies

CT26/HER2, 4T1.2/HER2, TC-1 and MC32 tumor cells were injected subcutaneously (s.c.) into the flank of BALB/c or C57BL6 mice. The tumor cells were grown in cDMEM or cRPMI (400 μg G418/ml), washed 2 times with PBS (for the CT26/HER2, TC-1 and MC32 cells) or with DMEM (for the 4T1.2/HER2 cells) and injected into mice in 100 μl of PBS or DMEM per mouse. The mice were monitored twice per week for tumor growth. Tumor growth was measured in mm using a caliper and was recorded as the mean diameter {longest surface length (a) and width (b), (a + b)/2}.

### Enzyme-linked immunosorbent assay (ELISA)

ELISAs to detect HER2-specific IgG were performed as previously described [45], except that recombinant HER2 protein (1 μg/ml in PBS) was used as a coating antigen. In particular, for the determination of the relative levels of HER2-specific immunoglobulin G (IgG) subclasses, anti-murine IgG1, IgG2a, IgG2b and IgG3 antibodies, which were conjugated with horseradish peroxidase (HRP) (Zymed, San Francisco, CA), were substituted for anti-murine IgG-HRP. Recombinant HER2 protein was purchased from Sino Biological Inc. (China).

### Preparation of splenocytes

Spleen was aseptically removed and physically broken for single cell suspension. Then, red blood cells were lysed by using red blood cell lysis buffer (Sigma-Aldrich, St. Louis, MO). The cells were filtered through a sieve (70 μm). Finally, splenocytes were washed 2 times with cRPMI media.

### IFN-γ assay

A 1 ml aliquot containing 6 × 10^6^ splenocytes was added to each well of 24-well plates containing 1 μg of a series of HER2 class I peptides (HER2_63–71_, HER2_342–350_, HER2_440–448_, HER2_553–561_, HER2_780–788_, HER2_907–915_), E7 class I peptides and CEA class I peptides. The HER2 class I peptides (HER2_63–71_, TYLPTNASL; HER2_342–350_, CYGLGMEHL; HER2_369–377_, KIFGSLAFL; HER2_440–448_, AYSLTLQGL; HER2_553–561_, EYVNARHCL; HER2_780–788_, PYVSRLLGI; HER2_907–915_, SYGVTVWEL), E7 peptides (RAHYNIVTF), CEA peptides (CGIQNSVSA) and Trp2 peptides (SVYDFFVWL) were purchased from Peptron, Taejon, Korea. After 2 days of incubation at 37°C in 5% CO_2_, cell supernatants were isolated and used to analyze IFN-γ levels. The analysis was performed using a commercial cytokine kit (BD Biosciences, San Jose, CA) and by adding the extracellular fluid samples to IFN-γ-specific ELISA plates. For the detection of systemic IFN-γ levels, mice were bled to collect serum samples. One hundred μl of each sample was added to IFN-γ-specific ELISA plates as described above.

### *In vivo* CTL lytic activity assay

One fraction of splenocytes was pulsed with 5 μg of HER2 peptides (HER2_63–71_ and HER2_342–350_) in cRPMI media for 60 min at 37°C, while the other fraction was left un-pulsed. To generate peptide-pulsed cells with high carboxyfluorescein diacetate succinimidyl ester (CFSE), the peptide-pulsed splenocytes were incubated with 20 μM CFSE in RPMI (2.5% FBS) for 15 min. The un-pulsed cells were instead incubated with 2.5 μM CFSE in RPMI (2.5% FBS) for 15 min to generate non-peptide-pulsed cells with low CFSE. The cells were then washed 3 times with PBS to remove unbound CFSE. Finally, an equal number of pulsed and un-pulsed cells (a total of 2 × 10^7^ cells/0.4 ml/mouse) were injected intravenously into the tested mice. After 48 h, the mice were sacrificed and the spleens were collected. After lysing the red blood cells, the splenocytes were analyzed directly for the two cell populations with CFSE staining (CFSE low versus CFSE high) using a flow cytometer (BD Biosciences). The percentage of lysed cells (%lysis) was calculated as 100 × {1− (*r*_unprimed_/*r*_primed_)}. The ratio (*r*) was calculated as %CFSE^low^/%CFSE^high^.

### *In vivo* depletion of CD8+ T and NK cells

Anti-CD8 IgGs (100 μg) or anti-asialo GM1 antibodies (200 μl) were administered intraperitoneally (i.p.) on the indicated days. A hybridoma cell line (clones 2.43) was purchased from the American Type Culture Collection (Manassas, VA), and anti-CD8 IgG was obtained as previously described [44]. Control rat IgG was purchased from Sigma-Aldrich. Anti-CD8 IgG treatment resulted in more than 98% depletion of CD8+ T cells at 7 days following antibody injection. Anti-asialo GM1 rabbit antibodies were purchased from Wako Pure Chemical, Osaka, Japan. Anti-asialo GM1 rabbit antibodies were diluted in accordance with the manufacturer's protocol and injected in 200 μl of PBS (containing the diluted anti-asialo GM1 Abs) per mouse. This Ab treatment resulted in 80–90% depletion of NK cells (expressing a CD3-/CD49b+ phenotype) at 5 days following antibody injection.

### Construction of CD80 plasmid DNA vectors and DNA transfection

Mouse CD80 genes were purchased from Sino Biological Inc., China. To generate CD80 expression vectors, CD80 genes were amplified using a pair of primers (forward primer, 5′-CGGAATTCATGGCTTGC AATTGTCAGTTG-3′ and reverse primer, 5′-CCGCTC GAGCTAAAGGAAGACGGTCTGTT-3′). The amplified DNA was digested with Eco RI and Xho I. The resulting DNA fragments were cloned into the Eco RI and Xho I sites of pcDNA3.1 (Invitrogen, Waltham, MA) and then cut with Kpn I and Xho I. The resulting Kpn I and Xho I DNA fragment was cloned into the Kpn I and Xho I sites of pCEP4 (Invitrogen), generating pCEP4-CD80. Tumor cells were transfected with 2 μg of pCEP4-CD80 using JetPEI™ transfection reagents (Polyplus-Transfection Inc., New York). One day after DNA transfection, 350 μg/ml hygromycin B was added to the cells. Hygromycin B-resistant cell colonies were selected and then tested for their CD80 expression status via fluorescence-activated cell sorting (FACS) analysis.

### FACS analysis

Five × 10^5^ tumor cells were treated at 4^°^C for 30 min with phycoerythrin (PE)-labeled Abs specific for CD80, CD86 and CD40, as well as fluorescein isothiocyanate (FITC)-labeled Abs specific for MHC class I (H-2K^d^) for FACS analysis. This procedure was conducted in parallel with a PE/FITC-labeled isotype control Ab treatment. For the detection of human HER2 expression, 2 μl of serum from mice that had been injected twice with 50 μg of HER2 DNA vaccine via intramuscular (IM)-electroporation (EP) was used to supply the primary antibodies. This was followed by a reaction with FITC-labeled anti-mouse IgG. The PE and FITC Abs were purchased from BD Biosciences. For preparation of immune cells from tumor, TDLN and spleen, tumor-bearing mice were sacrificed and each tissue was obtained. In particular, tumors were cut into small pieces in DMEM containing 1.5 mg/ml of collagenase type IV and 10 μg/ml of DNase, and then incubated at 37°C for 3 h. The tumor cell suspensions were applied to a 70 mm cell strainer. The collected cells were pretreated for 10 min with anti-CD16/32 (Fc blocker) and then stained with allophycocyanin-conjugated CD45 and PE-conjugated anti-CD11c, anti-CD11b, anti-CD4, anti-CD8 and anti-CD49b (clone DX5) as well as FITC-conjugated anti-Gr-1, anti-33D1 (DC marker) and anti-CD3. For intracellular staining, the cells were reacted with FITC-conjugated anti-CD4 antibodies. After washing, the cells were fixed and permeabilized with the Cytofix/Cytoperm kit (BD Biosciences). Intracellular staining was performed using PE-conjugated anti-FoxP3 antibodies. The anti-CD16/32, allophycocyanin, PE and FITC-conjugated Abs were purchased from BioLegend (San Diego, CA). In each step, the cells were washed twice with washing buffer (PBS+1% FBS). Finally, the cells were analyzed using a flow cytometer (BD Biosciences).

### Depletion of MDSCs by gemcitabine and anti-Gr-1 treatment

For depletion of MDSCs, the animals were treated i.p. with anti-Gr-1 Abs at a dose of 250 μg/mouse in 200 μl PBS as previously described [33]. Anti-Gr-1 Abs were purchased from Bio X Cells (West Lebanon, NH). Control rat immunoglobulin G (IgG) was purchased from Sigma-Aldrich. For gemcitabine treatment, animals were treated i.p. with gemcitabine (Gemzar^®^, Lilly) twice weekly (at 3 day intervals) at a dose of 75 μg/g of body weight as we previously described [33].

### IM-EP

For IM-EP delivery, mice were injected intramuscularly with 50 μg of HER2 DNA vaccines (pVAX1-HER2) per mouse in a final volume of 50 μl of PBS using a 31-gauge needle. The injections were followed by EP at 0.2 volts for 4 sec using Cellectra^®^ of VGX International Inc./Inovio in accordance with the manufacturer's protocol. The HER2 DNA vaccine, which coded for an extracellular region of the human HER2 protein, was kindly provided by W.Z. Wei (Wayne State University, Detroit, MI). Plasmid DNA was produced in bacteria and purified using endotoxin-free Qiagen kits according to the manufacturer's protocol (Qiagen, Valencia, CA).

### Statistical analysis

Statistical analysis was performed by one-way ANOVA using the SPSS 17.0 software program. The values of the experimental groups were compared with the values of the control group. Any *p* values < 0.05 were considered to be significant.

## Abbreviations

APCs: antigen presenting cells
CEA: carcinoembryonic antigen
CFSE: carboxyfluorescein diacetate succinimidyl ester
DCs: dendritic cells
ELISA: enzyme-linked immunosorbent assay
EP: electroporation
FACS: fluorescence-activated cell sorting
FBS: fetal bovine serum
FITC: fluorescein isothiocyanate
HER2: human epidermal growth factor receptor 2
HRP: horseradish peroxidase
IM: intramuscular
i.p.: intraperitoneally
MDSCs: myeloid-derived suppressor cells
PBS: phosphate-buffered saline
PD: programmed death
PE: phycoerythrin
s.c.: subcutaneously
TDLNs: tumor-draining lymph nodes
